# Lymphoma-Associated Bone Marrow Hemophagocytosis (LA-BMHPC): A Retrospective, Single-Center Study of 67 Patients

**DOI:** 10.7150/ijms.115901

**Published:** 2026-01-01

**Authors:** Feiyang Zong, Renjie Hua, Meng Dong, Xudong Zhang, Honghan Qiao, Sijun Zhang, Yukai Duan, Qingjiang Chen

**Affiliations:** 1 Department of Oncology, The First Affiliated Hospital, Zhengzhou University, Zhengzhou 450052, China.; 2 Henan Academy of Innovations in Medical Science, Zhengzhou 450052, China.

**Keywords:** lymphoma, hemophagocytosis, hemophagocytic lymphohistiocytosis, treatment, prognosis

## Abstract

**Objective:** To investigate the clinical characteristics, management strategies, and prognostic outcomes of patients with lymphoma-associated bone marrow hemophagocytosis (LA-BMHPC).

**Methods:** This retrospective, single-center cohort study enrolled patients diagnosed with LA-BMHPC between June 1, 2020, and June 30, 2023. We analyzed the clinical characteristics, treatment approaches (hemophagocytic lymphohistiocytosis [HLH]-directed therapy alone [HT], lymphoma-directed therapy alone [LT], simultaneous HLH and lymphoma therapy [HLT], no specific treatment [NST]), and overall survival (OS). Patients meeting the inclusion criteria (n=67) were categorized based on the HLH-2004 criteria into lymphoma-associated hemophagocytic lymphohistiocytosis (LA-HLH; n=50) and lymphoma-associated hemophagocytosis without fulfilling the HLH criteria (LA-HPC; n=17). Survival was monitored until November 30, 2023.

**Results:** The median overall survival (OS) of the entire LA-BMHPC cohort (n=67) was 3 months (range, 0-40 months), with 3-month, 1-year, and 3-year OS rates of 44.6%, 31.0%, and 22.7%, respectively. Significant differences between LA-HLH and LA-HPC groups were observed in the prevalence of fever, cytopenias (≥2 lineages), hypofibrinogenemia, hyperferritinemia and hypoalbuminemia (all P < 0.05). Receiver operating characteristic analysis identified elevated triglyceride and soluble CD25 levels as strong predictors of progression to LA-HLH, with optimal predictive cut-offs of 1.405 mmol/L and 1352.74 U/L, respectively. Multivariate Cox regression analysis revealed that LDH (hazard ratio [HR] 5.991,95% confidence interval [CI], 1.401—25.614; P=0.016), being treatment-naïve at the time of LA-BMHPC diagnosis (HR 2.537, 95% CI, 1.398—4.604; P=0.002), and treatment strategy (overall P=0.001) were independent prognostic factors. Compared to NST, both LT (HR 0.138, 95% CI, 0.046—0.414; P<0.001) and HLT (HR 0.117, 95% CI, 0.069—0.453; P<0.001) were associated with a significantly better survival benefit, whereas HT alone was not (HR 0.450, 95% CI, 0.172—1.180; P=0.104). Patients who received any form of lymphoma-directed therapy (LT or HLT) had significantly better OS than patients who did not (HT or NST; HR = 0.301, 95% CI, 0.160—0.568; P < 0.001). Patients with LA-HPC exhibited a significantly better OS (median, 17 months;1-year rate, 63.7%) than those with LA-HLH (median, 2 months;1-year rate, 20.6%; P=0.015).

**Conclusions:** LA-BMHPC defines a spectrum of diseases ranging from a high-risk precursor state (LA-HPC) to fulminant LA-HLH. Progression to LA-HLH is associated with fever, cytopenias (≥2 lineages), hypofibrinogenemia, hyperferritinemia, hypoalbuminemia, and elevations in triglyceride (≥1.405 mmol/L) or soluble CD25 (≥1352.74 U/L) levels. Effective treatment of the underlying lymphoma is the most critical determinant of survival. An integrated strategy (HLT) represents a rational approach, potentially serving as a “bridge” by controlling hyperinflammation to enable definitive anti-lymphoma therapy.

## Introduction

Hemophagocytic lymphohistiocytosis (HLH) is a severe hyperinflammatory syndrome caused by the pathological activation of immune cells, including cytotoxic T -lymphocytes and macrophages, leading to a cytokine storm and potential multi-organ failure 1. While primary (familial) forms exist, often due to genetic mutations affecting cytotoxic pathways, secondary HLH triggered by infections, autoimmune diseases, or malignancies is more prevalent in adults 1, 2. Malignancy-associated HLH (M-HLH), particularly lymphoma-associated HLH (LA-HLH), represents a considerable clinical challenge 3, 4. LA-HLH is associated with an aggressive disease course and carries a particularly poor prognosis, with reported median survival often measured in months 3.

Hemophagocytosis (HPC), the engulfment of hematopoietic cells by activated macrophages/histiocytes, is a characteristic pathological finding frequently observed in the bone marrow (BMHPC) 5, 6. LA-HLH often presents with advanced-stage lymphoma (Ann Arbor stage III/IV) and bone marrow infiltration 3. The Diagnosis of LA-HLH is complicated by the overlap of symptoms with an underlying malignancy, infections, or treatment effects 3. Standard diagnostic criteria such as the HLH-2004 were primarily developed for pediatric populations and may have limitations in the context of adult M-HLH 7, 8. Optimal management requires balanced treatment for both hyperinflammation and the underlying lymphoma; however, evidence-based protocols remain limited, especially for adults 3, 8. Conceptually, it is perilous to conflate the histological findings of hemophagocytosis with those of HLH. BMHPC is a pathological observation, and its clinical significance can range from a benign reactive state to a harbor of severe systemic disease 9. In contrast, HLH is a clinical syndrome defined by specific diagnostic criteria that reflect a systemic cytokine storm 10. Although the presence of BMHPC is neither sensitive nor specific for the diagnosis of HLH, we hypothesized that, in the specific context of lymphoma, the emergence of BMHPC may signify a critical, potentially reversible, early phase of hyperinflammation 4, 5, 11. This phase may precede the destructive systemic dysfunction that is required to fulfill the HLH-2004 criteria.

Therefore, the objective of this study was not to imply that all cases of lymphoma-associated bone marrow hemophagocytosis (LA-BMHPC) inevitably progressed to LA-HLH. Rather, by retrospectively analyzing a cohort of patients spanning the spectrum from a histological manifestation (defined here as lymphoma-associated HPC, LA-HPC) to complete clinical syndrome (LA-HLH), this study aimed to identify clinical and laboratory parameters predictive of progression to fulminant LA-HLH.

A further objective was to evaluate whether early preemptive intervention, particularly at the LA-HPC stage before the full HLH criteria are met, improved patient prognosis. Through this approach, we sought to provide an evidence base to guide clinicians toward timely and effective interventions when confronted with sentinel findings of BMHPC in patients with lymphoma.

## Materials and Methods

### Patient Cohort

Clinical data were retrospectively collected from patients aged ≥18 years diagnosed with LA-BMHPC at the First Affiliated Hospital of Zhengzhou University between June 1, 2020, and June 30, 2023.

### Study Design and Data Collection

Sixty-seven patients with LA-BMHPC fulfilling the inclusion criteria were identified. Based on the HLH-2004 diagnostic criteria 12, the patients were stratified into two groups: lymphoma-associated hemophagocytic lymphohistiocytosis (LA-HLH, n=50) and lymphoma-associated LA-HPC without fulfilling the HLH criteria (LA-HPC, n=17). Data obtained included general information (age, sex), clinical manifestations (fever, splenomegaly), lymphoma characteristics (subtype, Ann Arbor Stage, Eastern Cooperative Oncology Group Performance Status [ECOG PS], International Prognostic Index [IPI], number of extranodal lesions, other tumor burden[bone marrow involvement, number of involved nodal regions]), laboratory parameters (complete blood count, liver function tests, lactate dehydrogenase [LDH], coagulation profile, ferritin, bone marrow cytology), time to diagnosis, treatment administered, and survival status. The follow-up was conducted until November 30, 2023, with the primary endpoint being death from any cause. Overall survival (OS) was defined as the time from LA-BMHPC diagnosis to death or the date of the last follow-up. All study procedures adhered to the relevant ethical guidelines and regulations.

### Definitions and Criteria

Inclusion Criteria**:** 1) Age ≥18 years;2) Evidence of HPC on bone marrow biopsy and/or aspirate smears;3) Histologically confirmed diagnosis of lymphoma according to established international criteria.

HLH Diagnosis**:** Diagnosis of HLH required meeting the HLH-2004 criteria: fulfillment of ≥5 of the 8 clinical and laboratory criteria (fever ≥38.5°C, splenomegaly, cytopenias affecting ≥2 lineages [hemoglobin <9 g/dL, platelets <100 × 10⁹/L, neutrophils <1.0 × 10⁹/L], hypertriglyceridemia [fasting ≥3.0 mmol/L] and/or hypofibrinogenemia [≤1.5 g/L], HPC in tissues, low/absent NK-cell activity, hyperferritinemia [≥500 µg/L], elevated soluble CD25 [≥2400 U/mL])^12^ . Patients with confirmed lymphoma and BMHPC who met the <5 HLH-2004 criteria were diagnosed with LA-HPC.

Treatment Strategies: Initial treatment strategies for LA-BMHPC were classified as follows: HLH-directed therapy alone (HT; e.g., corticosteroids, etoposide); lymphoma-directed therapy alone (LT; e.g., chemotherapy, targeted therapy); simultaneous HLH- and lymphoma-directed therapy (HLT); or no specific therapy, consisting of only palliative/supportive care (NST).

### Statistical Analysis

Statistical analyses were performed using the SPSS version 26.0 software (IBM Corp, Armonk, NY, USA). Survival plots were generated using GraphPad Prism version 8.3.0 (GraphPad Software, San Diego, CA, USA). Categorical variables were compared using the chi-squared test or Fisher's exact test, as appropriate. Quantitative variables were compared using the independent two-sample t-test or Mann—Whitney U test, depending on the normality of the data distribution. Receiver operating characteristic (ROC) curves were used to evaluate the diagnostic performance of continuous variables for binary outcomes. Univariate and multivariate survival analyses were performed using Cox proportional hazards models to identify the independent prognostic factors. Survival curves were estimated using the Kaplan—Meier method and compared using the log-rank test. The threshold for statistical significance was defined as a two-sided P-value < 0.05.

## Results

### Baseline Characteristics

A total of 67 patients with LA-BMHPC were included, comprising 40 (59.7%) men and 27 (40.3%) women, with a median age of 50 years (range, 18-78 years). The lymphoma subtypes included Hodgkin lymphoma (n=1), NK/T-cell lymphoma (n=28), B-cell lymphoma (n=26), and T-cell lymphoma (n=12). Common laboratory abnormalities at diagnosis included elevated LDH (58/67,86.6%), hypoalbuminemia (54/67,80.6%), and hyperferritinemia (53/67,79.1%). A comparison between the LA-HLH (n=50) and LA-HPC (n=17) groups is presented in Table 1. Compared to the LA-HPC group, patients who progressed to LA-HLH exhibited a significantly higher incidence of key diagnostic features, including fever, cytopenias (≥2 lineages), hypofibrinogenemia, hypoalbuminemia, and hyperferritinemia (all P<0.05). Consistent with these findings, the LA-HLH cohort also demonstrated significantly lower median platelet and fibrinogen levels, along with markedly elevated levels of triglycerides (TG), ferritin, LDH, and soluble interleukin-2 receptor (sCD25) (all P<0.05). Additionally, the time to diagnosis was significantly shorter in the LA-HLH group (P=0.012). Notably, the two groups did not differ significantly in terms of baseline demographics (age and sex), lymphoma characteristics (subtype, Ann Arbor Stage, ECOG PS, IPI score, and number of extranodal lesions), or prior treatment status (all P>0.05). Furthermore, no significant differences were observed in other laboratory parameters, including hemoglobin (Hb), absolute neutrophil count, or markers of liver function such as alanine aminotransferase, aspartate aminotransferase, and bilirubin levels (all P>0.05).

**Figure 1:**
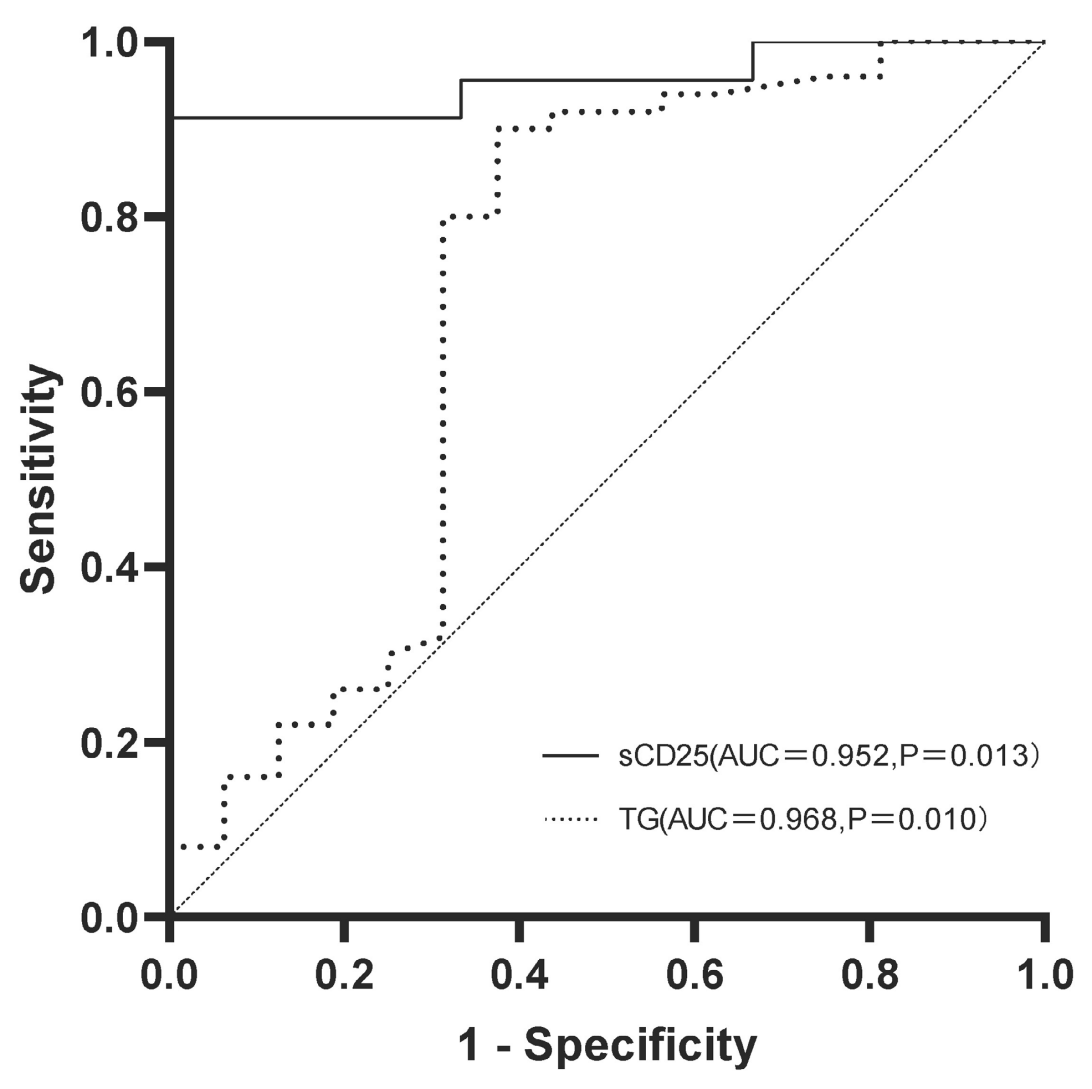
ROC curves of sCD25 and TG for predicting progression to LA-HLH. The AUC were 0.952 for sCD25 and 0.968 for TG. Abbreviations: AUC: area under the curve; LA-HLH: lymphoma-associated hemophagocytic lymphohistiocytosis; ROC: receiver operating characteristic; sCD25: soluble CD25 (interleukin-2 receptor); TG: triglycerides.

To evaluate the utility of key laboratory parameters in predicting progression to LA-HLH, ROC curve analysis was performed. TG and sCD25 levels demonstrated the highest predictive value (Figure 1). The area under the curve for sCD25 was 0.952 (95% confidence interval [CI]:0.865-1.000; P=0.013), with an optimal predictive cutoff of 1352.74 U/L. TG yielded the highest performance with an area under the curve of 0.968 (95% CI: 0.898-1.000; P=0.010) and an optimal predictive cutoff of 1.405 mmol/L. Interestingly, while median TG levels as a continuous variable were significantly higher in the LA-HLH group (P=0.012), the proportion of patients meeting the formal HLH-2004 diagnostic criterion for hypertriglyceridemia (fasting TG ≥3.0 mmol/L) did not differ significantly between the groups (P=0.571). This discrepancy, supported by our ROC analysis identifying a much lower optimal predictive cut-off, suggests that the established high-level diagnostic threshold may lack sensitivity for identifying progression risk in this specific patient population.

### Treatment Modalities and Outcomes

Among the 67 patients, treatment strategies were as follows: HT (n=13), LT (n=12), HLT (n=34), and NST (n=8). The median OS varied by treatment group as follows: 1 month (range, 0-26 months) for HT, 12 months (range, 1-22 months) for LT, 4 months (range, 1-40 months) for HLT, and 1 month (range, 0-2 months) for NST. At the follow-up deadline (November 30, 2023), 16 (23.9 %) patients were still alive. Among the survivors, 11 received HLT (median follow-up, 23 months), 3 received LT (follow-up 5, 6 and 22 months), 1 received HT (follow-up 10 months), and 1 received NST (follow-up 2 months).

Among the four treatment cohorts, patients in the HT group (n=13) were predominantly individuals who had already progressed to overt LA-HLH before therapy (n=12, 92.3%). The treatment for this group primarily consisted of an HLH-94-based protocol (etoposide and dexamethasone) in 12 patients, while one patient received a DEP-based regimen (liposomal doxorubicin, etoposide, and methylprednisolone) plus cyclosporine. Concurrent anti-lymphoma therapy is often prevented by significant organ failure. The prognosis for this cohort was poor, with 11 patients (84.6%) surviving for 3 months or less. In contrast, most patients in the LT group (n=12) did not meet the full HLH diagnostic criteria at the time of lymphoma-directed therapy initiation (n=9, 75.0%). Similarly, in the largest cohort, the HLT group (n=34), most patients (n=27, 79.4%) also progressed to LA-HLH before starting the combined therapy. The therapeutic regimens in this group varied and included HLH-94 plus chemotherapy with or without targeted/immunotherapy (n=24), DEP-based regimens (n=5), and HLH-04-based protocols (n=3). Notably, one patient in this group underwent hematopoietic stem cell transplantation after receiving intensive multimodal therapy. Finally, the NST group (n=8) consisted largely of patients who had already progressed to LA-HLH (n=7, 87.5%). These individuals generally experience early mortality, often due to severe circulatory failure, or a decision to decline active treatment in favor of supportive care.

### Survival Analysis and Prognostic Factors

**Figure 2:**
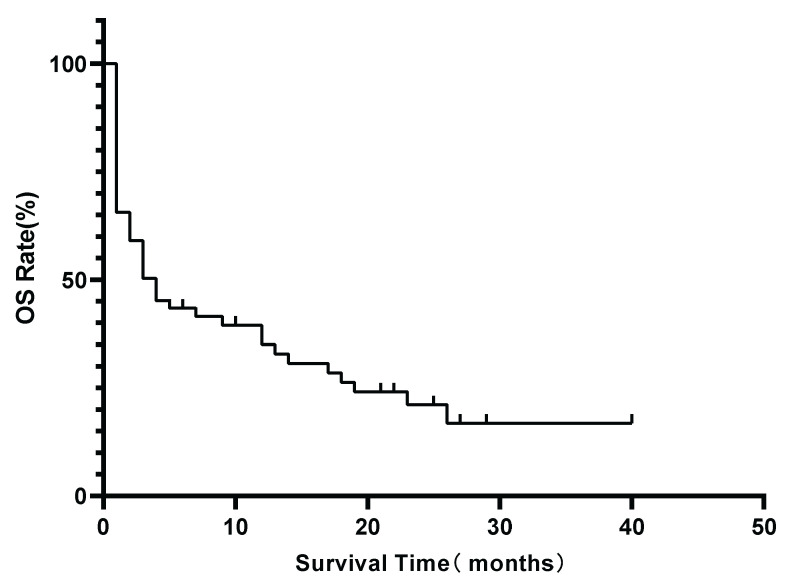
Kaplan-Meier estimate of overall survival (OS) for the entire lymphoma-associated bone marrow hemophagocytosis(LA-BMHPC) cohort (n=67).

All 67 patients were included in survival analysis. During the follow-up period, 51(76.1%) patients died. The median OS for the entire cohort was 3 months (range, 0-40 months). The estimated 1-year and 3-year OS rates were 31.0% and 22.7%, respectively (Figure 2).

**Figure 3:**
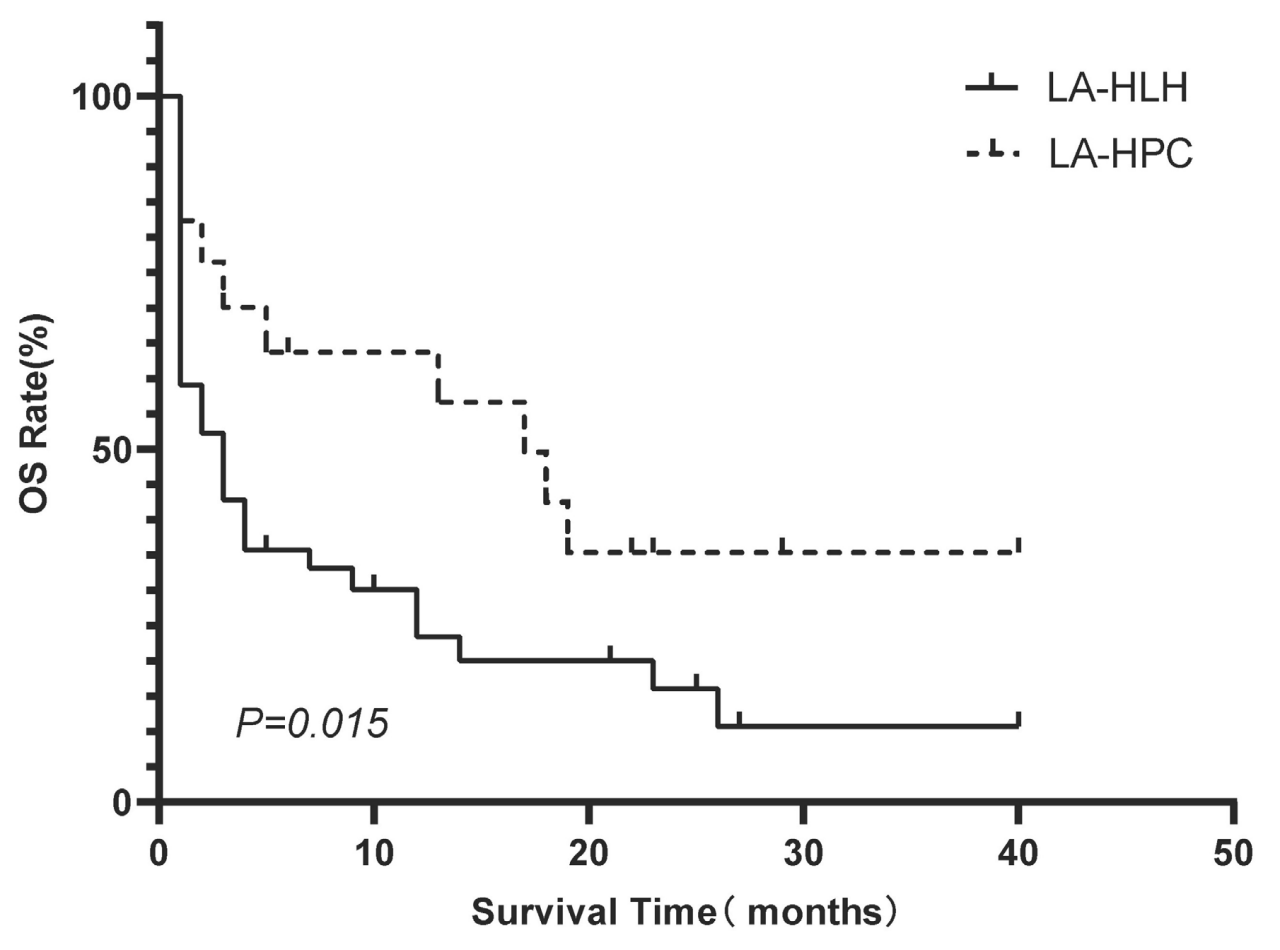
Kaplan-Meier comparison of OS between the LA-HLH (n=50) and LA-HPC (n=17) groups. Survival distributions were significantly different (log-rank P=0.015). Abbreviations: HLH: hemophagocytic lymphohistiocytosis; LA-HLH: lymphoma-associated hemophagocytic lymphohistiocytosis; LA-HPC: lymphoma-associated hemophagocytosis without fulfilling HLH criteria; OS: overall survival.

Patients in the LA-HPC group (n=17) had significantly better survival outcomes than those in the LA-HLH group (n=50). The median OS was 17 months for patients with LA-HPC and 2 months for patients with LA-HLH (P=0.015). The 1-year OS rates were 63.7% and 20.6% in the LA-HPC and LA-HLH groups, respectively (Figure 3).

Univariate analysis identified several factors significantly associated with OS: LDH level >245 U/L, initial treatment status (treatment-naïve vs. prior treatment), and treatment strategy (HT, LT, HLT, and NST) (all P<0.05, Table 2).

**Figure 4:**
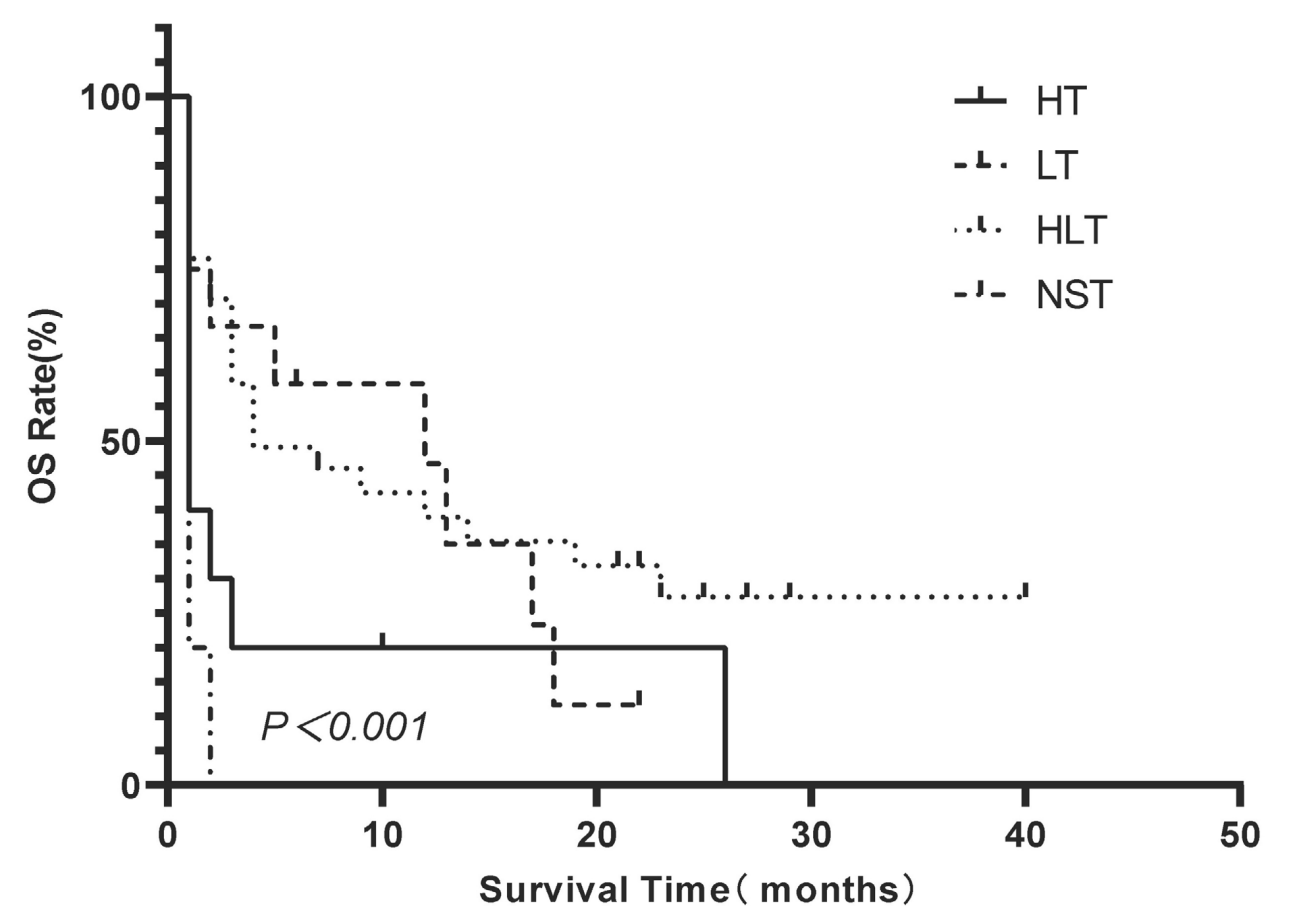
Kaplan-Meier comparison of OS among LA-BMHPC patients stratified by treatment strategy. Survival distributions differed significantly among the groups (log-rank P≤0.001). Abbreviations: HLH: hemophagocytic lymphohistiocytosis; HT: HLH-directed therapy alone; LT: lymphoma-directed therapy alone; HLH: hemophagocytic lymphohistiocytosis; HLT: simultaneous HLH and lymphoma therapy; LA-BMHPC: lymphoma-associated bone marrow hemophagocytosis; NST: no specific treatment; OS: overall survival.

**Figure 5:**
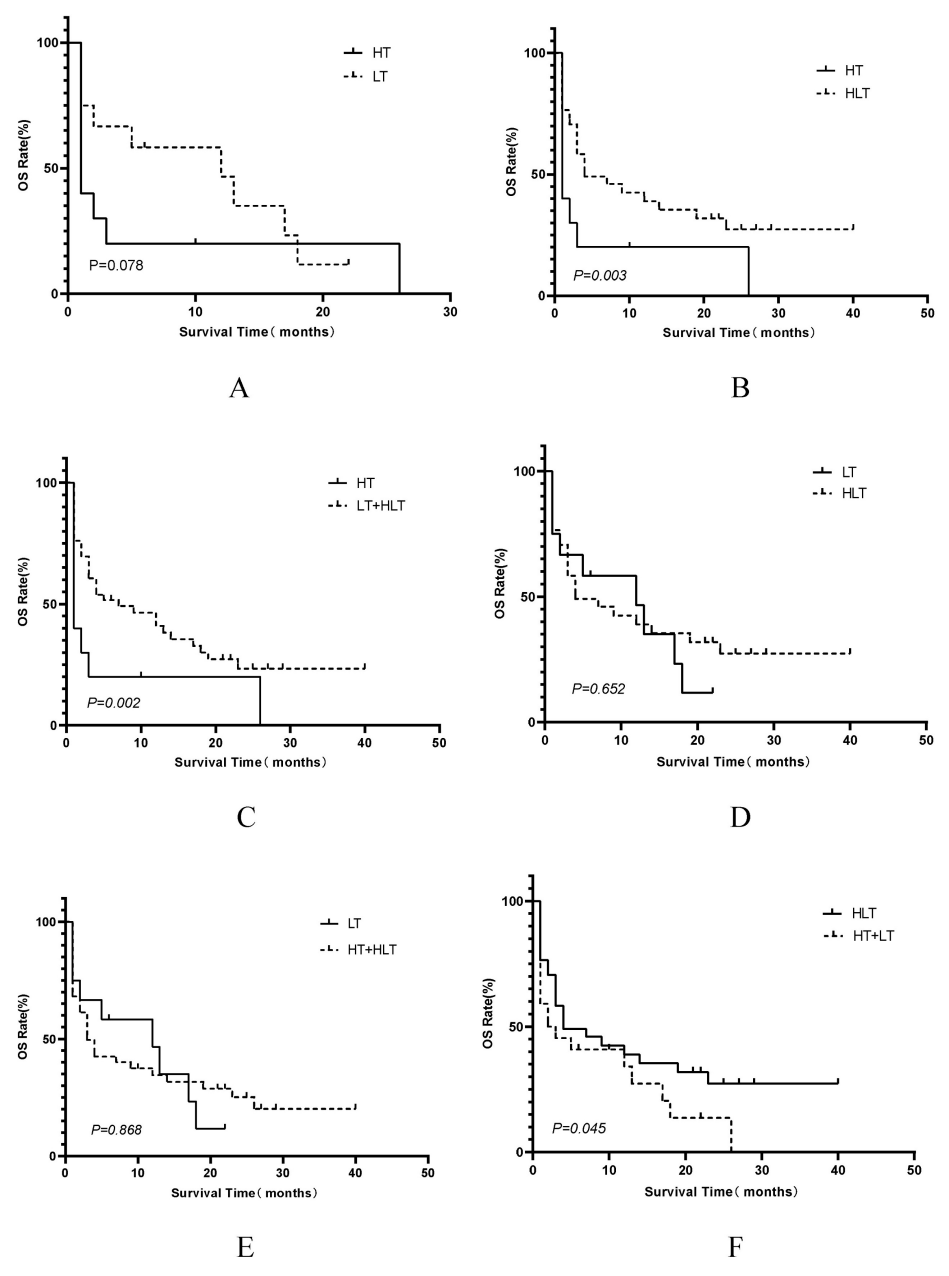
Kaplan-Meier comparisons of OS among different treatment strategy groups. Panels display OS comparisons between: (A) HT vs. LT; (B) HT vs. HLT; (C) HT vs. the combined LT+HLT group; (D) LT vs. HLT; (E) LT vs. the combined HT+HLT group; (F) HLT vs. the combined HT+LT group. Survival distributions were compared using the log-rank test. Abbreviations: HLH: hemophagocytic lymphohistiocytosis;HT: HLH-directed therapy alone; LT: lymphoma-directed therapy alone; HLH: hemophagocytic lymphohistiocytosis; HLT: simultaneous HLH and lymphoma therapy; OS: overall survival.

Multivariate Cox proportional hazards regression analysis confirmed that elevated LDH levels (hazard ratio [HR]=5.991, 95% CI 1.401—25.614; P=0.016), being treatment-naïve at the time of LA-BMHPC diagnosis (HR=2.537, 95% CI 1.398—4.604; P=0.002), and the treatment strategy employed (overall P=0.001) were independent predictors of poor prognosis (Table 2). Compared to NST, both LT (HR 0.138, 95% CI 0.046—0.414; P<0.001) and HLT (HR 0.117, 95% CI 0.069—0.453; P<0.001) were associated with a significant survival benefit. HT alone did not reach statistical significance (HR 0.450, 95% CI 0.172—1.180; P=0.104). To further dissect the prognostic contributions of the different therapeutic components, we conducted three separate multivariate Cox regression analyses, each adjusted for LDH levels and initial treatment status. These analyses revealed that receiving any form of lymphoma-directed therapy (Model B:HR 0.301, 95% CI 0.160—0.568; P<0.001) was independently associated with significantly improved prognosis. In contrast, neither HLH-directed therapy (Model A: HR 0.951, 95% CI 0.517-1.748; P=0.872) nor the combined HLT regimen (Model C: HR 0.630; 95% CI 0.357-1.111, P=0.110) showed a statistically significant independent survival benefit when compared to other strategies (Table 3). Kaplan-Meier analysis of the four treatment groups (Figure 4) showed a significant overall difference in survival (log-rank P<0.001). Pairwise comparisons (Figure 5) showed that HLT resulted in a significantly better OS than HT alone (P=0.003). Although the overall comparison between the LT and HLT groups was not statistically significant (P=0.652), the survival curves appeared to cross, indicating a potential non-proportional hazard scenario. LT alone was associated with a favorable short-term survival trajectory, whereas the integrated HLT approach was associated with superior survival probability at later time points.

**Figure 6:**
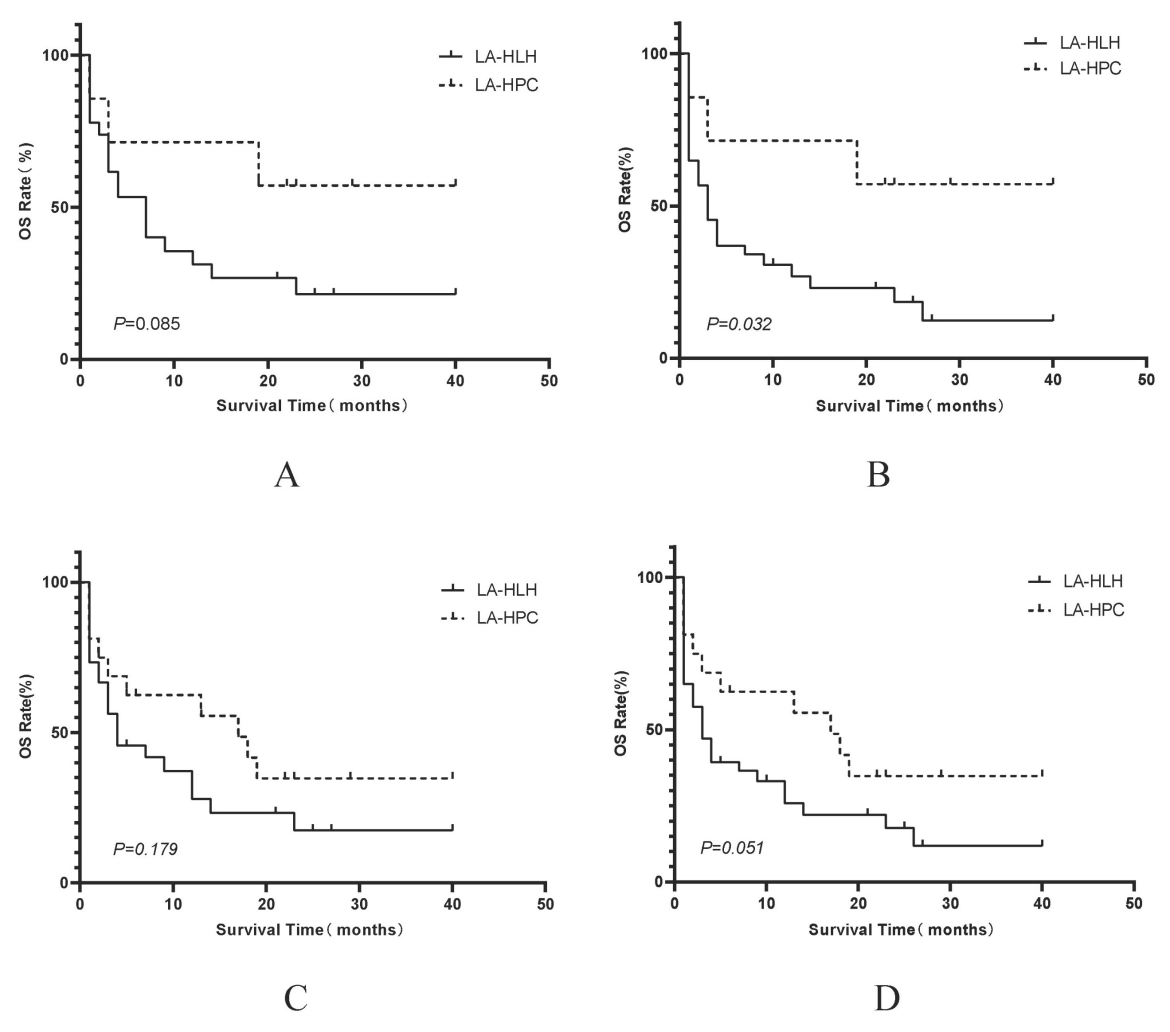
Kaplan-Meier comparisons of OS between LA-HPC and LA-HLH patient groups within specific treatment cohorts. Panels display OS comparisons between LA-HPC and LA-HLH patients among those who received: (A) HLT (n=7 LA-HPC vs. n=27 LA-HLH); (B) HLT or HT (n=7 LA-HPC vs. n=40 LA-HLH); (C) HLT or LT (n=16 LA-HPC vs. n=30 LA-HLH); (D) any active treatment (HLT, HT, or LT) (n=16 LA-HPC vs. n=43 LA-HLH). Survival distributions were compared using the log-rank test. Abbreviations: HLH: hemophagocytic lymphohistiocytosis; HLT: simultaneous HLH and lymphoma therapy; HT: HLH-directed therapy alone; LA-HLH: lymphoma-associated hemophagocytic lymphohistiocytosis; LA-HPC: lymphoma-associated hemophagocytosis without fulfilling HLH criteria; LT: lymphoma-directed therapy alone; OS: overall survival.

In the subgroup of patients who received HLT, the median overall survival for the LA-HPC group was 22 months, and it was 4 months in the LA-HLH group. Although a difference in survival was observed, it did not reach statistical significance according to the log-rank test (P=0.085; Figure 6A). Furthermore, analysis of outcomes based on HLH status at treatment initiation revealed that LA-HPC patients who received early intervention (HT or HLT) had significantly better survival than LA-HLH patients receiving the same treatments (HT or HLT) after fulfilling HLH criteria (P<0.05, Figure 6B).

## Discussion

We analyzed a cohort of patients with fulminant clinical syndrome (LA-HLH) across a spectrum of lymphoma-associated BMHPC, from a precursor state (LA-HPC) to the fulminant clinical syndrome (LA-HLH) 3, 13. Our research was predicated on the hypothesis that in patients with lymphoma, the histological finding of BMHPC serves as a critical sentinel event, heralding an evolving hyperinflammatory state that may precede irreversible, multi-organ failure. Our findings support this hypothesis and offer critical insights into clinical management.

The most striking finding was the significant prognostic relationship between LA-HPC and LA-HLH. Patients with established LA-HLH have a dismal prognosis, with a median OS of just 2.0 months, which is consistent with previous reports on this aggressive condition 1, 3. In contrast, patients diagnosed at the LA-HPC stage before meeting the full HLH criteria had a markedly better median OS of 17.0 months. This significant survival disparity highlights the existence of a critical “window of opportunity” for intervention. Our study operationalizes the concept of a precursor state, which has been described more amorphously in other contexts such as “subclinical macrophage activation syndrome (MAS)” 14, 15. Unlike these broader terms, LA-HPC is defined specifically within the lymphoma population by a concrete histological finding (BMHPC), and, as our data show, it is associated with quantifiable markers that predict progression. This provides a more precise framework for identifying patients in a clinically distinct, high-risk phase, where the cytokine storm may not be fully established and organ damage may still be reversible.

A central and robust finding of this study was that the administration of lymphoma-directed therapy is the most powerful determinant of survival. The adjusted multivariate analysis unequivocally showed that patients receiving either LT or HLT had a nearly 70% reduction in the risk of death compared with those who did not (HR 0.301, P<0.001). This finding strongly supports the guiding principle in the management of M-HLH: durable remission is contingent upon effective eradication of the underlying malignant trigger 8. While our analysis did not show a statistically significant independent survival benefit for HLT over other strategies in the adjusted model (P=0.110), Kaplan—Meier analyses suggested a more nuanced role. The superior outcomes observed with HLT compared with HT alone (P=0.003) suggest that the HLH-directed component of HLT acts as a crucial “bridge to therapy”. Suppression of the life-threatening cytokine storm with agents such as corticosteroids and etoposide stabilizes the patient, thereby allowing definitive anti-lymphoma therapy to be administered safely and effectively 1, 16-18. The debate on etoposide use in adults persists owing to toxicity concerns, but recent meta-analyses support its effectiveness in improving response rates and survival in adult HLH, reinforcing its potential value in this bridging role 16, 17.

The lack of a significant survival benefit for HLH-directed therapy as a standalone variable in our model likely reflects profound confounding by indication; patients whose condition is stable enough to receive anti-lymphoma treatment have an inherently better prognosis than those whose clinical status is so precarious that only supportive or HLH-directed therapies are feasible 4, 9. Similarly, the HLT strategy itself did not demonstrate an independent statistical advantage over the other therapeutic approaches in the adjusted multivariate analysis (Model C, P=0.110). This finding is likely attributable to profound confounding by indication, a well-described methodological challenge in non-randomized studies of critically ill patients 19. Clinically, patients who are most critically ill—those with established or impending multi-organ failure characteristic of fulminant LA-HLH—are precisely the population for whom intensive combined HLT is deemed necessary 1. Conversely, patients with a more stable clinical status and less severe inflammatory response (i.e., the LA-HPC group) are more likely to be candidates for LT alone 1. Our data clearly reflect this treatment allocation reality: 79.4% of HLT recipients had already progressed to LA-HLH, whereas 75.0% of LT recipients were treated at a more prognostically favorable LA-HPC stage. This inherent disparity in the baseline prognosis, in which the sickest patients are preferentially channeled into the HLT arm, creates a powerful confounding effect that can mask the true therapeutic benefits of the HLT strategy in standard regression models. Such biases are recognized as a major challenge in interpreting retrospective data on malignancy-associated HLH, in which the ability to tolerate a specific therapy may itself be a marker of better prognosis.

Prompt diagnosis of M-HLH remains challenging, as standard criteria such as HLH-2004 have not been validated for adults, and symptoms overlap with malignancy and its treatment 1. This has spurred the development of tools such as the HScore, and more specifically, the optimized HLH inflammatory (OHI) index for M-HLH 18, 20, 21. Our findings complemented this paradigm. The OHI, which uses cutoffs of ferritin >1000 ng/mL and sCD25 >3900 U/mL, was designed to identify established M-HLH with a high mortality risk 8, 18, 20. In contrast, our analysis identified key predictors of LA-HLH progression. Beyond the established HLH-2004 criteria components like fever, cytopenias and hypofibrinogenemia, we established predictive cutoffs for TG (≥1.405 mmol/L) and sCD25 (≥1352.74 U/L) that are lower than the formal diagnostic thresholds. This suggests that our markers do not simply predict risk but rather help define a clinically distinct, high-risk precursor state, the LA-HPC phase, that precedes the fulminant syndrome identified by the OHI index 20. This provides an evidence-base for a strategy of preemptive intervention targeting patients within a critical therapeutic window before an irreversible cytokine storm occurs.

The fact that baseline lymphoma characteristics (e.g., Ann Arbor stage and IPI score) did not differ between the LA-HPC and LA-HLH groups suggests that progression to fulminant HLH is driven more by the host's dysregulated immune response than by the tumor burden itself 1, 3, 6. Multivariate analysis also identified elevated serum LDH levels and treatment-naïve status at diagnosis as powerful independent predictors of poor survival. Elevated LDH is an established biomarker of tumor burden and tissue damage in both lymphoma and HLH 3, 22, 23. The counterintuitive finding that treatment-naïve status portends a worse prognosis is a key insight, challenging the assumption that these patients possess a greater physiological reserve. This finding suggests that LA-BMHPC is not a homogeneous event. In patients with relapsed or refractory disease, HPC may arise as a complication of tumor progression or therapy-induced immunosuppression 1, 8, 24. In contrast, its appearance in treatment-naïve patients often signals a fundamentally different and more aggressive disease biology from its inception 25. We propose conceptualizing this as an “HLH-phenotype lymphoma”. In these cases, hyperinflammatory syndrome is not merely a secondary complication but an intrinsic and core feature of the pathophysiology of malignancy 26. This is particularly characteristic of certain Epstein—Barr virus-associated T-cell and NK-cell lymphomas, where the neoplastic cells themselves are potent drivers of a catastrophic immune response, creating a state of T-cell activation comparable to that of primary HLH 6, 27, 28. This distinction has significant implications for clinical practice. Identifying an “HLH-phenotype lymphoma” at first presentation serves as a critical tool for immediate risk stratification, flagging patients with a devastatingly poor prognosis who require the most aggressive, integrated therapeutic strategies from the outset.

This study has limitations inherent to its retrospective, single-center design, including potential selection bias and limited sample size, which restrict subgroup analyses (e.g., by specific lymphoma subtypes) and generalizability. The heterogeneity of lymphoma subtypes (NK/T-cell, B-cell, Hodgkin) is a significant confounder, as prognosis and treatment responses vary widely 3, 8, 29. Although large reviews have confirmed roughly equal proportions of NK/T-cell lymphoma and B-cell lymphoma in LA-HLH cohorts, subtype-specific analyses in larger studies are crucial 3. The absence of a non-BMHPC lymphoma control group limits definitive conclusions about the independent prognostic impact of BMHPC itself versus the underlying aggressive lymphoma biology. Indeed, the histological finding of BMHPC itself might be considered a manifestation of this aggressive biology, as detailed in pathological studies 30. Future prospective studies should validate our proposed predictive markers, incorporate newer diagnostic tools like the OHI index, and explore the role of novel targeted agents, such as the interferon -γ inhibitor emapalumab or Janus kinase inhibitors like ruxolitinib, which may offer less toxic bridging strategies to definitive therapy 18, 31, 32.

LA-BMHPC represents a clinical spectrum ranging from a high-risk precursor state (LA-HPC) to a fulminant life-threatening syndrome (LA-HLH). The early recognition of BMHPC in lymphoma patients, particularly those presenting with fever, cytopenias (≥2 lineages), hypofibrinogenemia, hyperferritinemia and hypoalbuminemia, or elevations in TG and sCD25 above our identified predictive thresholds, provides a critical window for intervention. Our findings demonstrate that the single most important factor for improving the prognosis of this condition is the administration of effective lymphoma-directed therapy. Although controlling hyperinflammation is crucial, the ultimate goal is to eradicate malignant triggers. Therefore, a prompt combined therapeutic strategy that targets both HLH-driven hyperinflammation and the underlying lymphoma (HLT) is a rational approach. The HLH-directed component serves as an essential bridge, stabilizing the patient and mitigating organ damage to allow safe and timely delivery of definitive antilymphoma treatment. Initiating such aggressive integrated therapy at the LA-HPC stage before the full criteria for LA-HLH are met offers the greatest opportunity to alter the devastating natural history of this condition.

## Figures and Tables

**Table 1:** Baseline Characteristics of LA-HLH and LA-HPC Patients

Parameters	LA-HLH (50 cases)	LA-HPC (17 cases)	P value
Sex			0.932
Men	30(60.00%)	10(58.82%)	
Women	20(40.00%)	7 (41.18%)	
Age(years)			0.411
≤60	34(68.00%)	14(82.35%)	
>60	16(32.00%)	3 (17.65%)	
Pathological type			0.931
Hodgkin lymphoma and B cell lymphoma^a^	21(42.00%)	6 (35.29%)	
NK/T cell lymphoma	20(40.00%)	8 (47.06%)	
T cell lymphoma	9 (18.00%)	3 (17.65%)	
ECOG PS			0.825
0-1	41(82.00%)	15(88.24%)	
≥2	9 (18.00%)	2 (11.76%)	
Ann Arbor Stage			0.977
I-II	8 (16.00%)	2 (11.76%)	
III-IV	42(84.00%)	15(88.24%)	
Number of extranodal lesions			0.069
0-1	16(32.00%)	1 ( 5.88%)	
≥2	34(68.00%)	16(94.12%)	
IPI^b^			0.895
Low-intermediate risk	5 (10.00%)	1 ( 5.88%)	
High-intermediate risk	15(30.00%)	5 (29.41%)	
High risk	30(60.00%)	10(58.82%)	
Bone marrow involvement			0.716
Yes	29(58.00%)	9 (52.94%)	
No	21(42.00%)	8 (47.06%)	
Number of involved nodal regions			0.231
<4	23(46.00%)	5 (29.41%)	
≥4	27(54.00%)	12(70.59%)	
Fever^c^			**<0.001**
Yes	42(84.00%)	5 (29.41%)	
No	8 (16.00%)	12(70.59%)	
Splenomegaly			0.229
Yes	29(58.00%)	7 (41.18%)	
No	21(42.00%)	10(58.82%)	
Hb, Mean (SD), g/L	83.41±17.35	92.39±19.09	0.077
PLT, Median (IQR), × 10⁹/L	38.50(21.75-62.50)	98.00(51.00-169.50)	**<0.001**
ANC, Median (IQR), × 10⁹/L	2.24(1.29-4.69)	3.68(1.86-7.68)	0.142
Cytopenias^d^			**<0.001**
Yes	41(82.00%)	6 (35.29%)	
No	9 (18.00%)	11(64.71%)	
TG, Median (IQR), mmol/L	2.17(1.76-2.80)	1.40(1.17-2.69)	**0.012**
Hypertriglyceridemia^e^			0.571
Yes	11(22.00%)	2 (11.76%)	
No	39(78.00%)	15(88.24%)	
FIB, Median (IQR), g/L	1.85(1.03-2.46)	2.36(1.90-3.60)	**0.007**
Hypofibrinogenemia^f^			**0.021**
Yes	20(40.00%)	1 ( 5.88%)	
No	30(60.00%)	16(94.12%)	
FER, Median (IQR), ng/mL	3296.05(1845.82-10764.25)	1090.75(407.28-2825.60)	**0.002**
Hyperferritinemia^g^			**<0.001**
Yes	46(92.00%)	7 (41.18%)	
No	4 ( 8.00%)	10(58.82%)	
sCD25, Median (IQR), U/mL	7281.96(2572.31-10000.00)	665.28(374.94-924.65)	**0.005**
ALT(U/L)			0.442
>40	23(46.00%)	6 (35.29%)	
≤40	27(54.00%)	11(64.71%)	
AST(U/L)			0.099
>40	32(64.00%)	7 (41.18%)	
≤40	18(36.00%)	10(58.82%)	
LDH, Median (IQR), U/L	852.50(591.00-1384.75)	368.00(258.00-1087.00)	**0.009**
LDH(U/L)			0.203
>245	46(92.00%)	13(76.47%)	
≤245	4 ( 8.00%)	4 (23.53%)	
DBIL(μmol/L)			0.361
>10	27(54.00%)	7 (41.18%)	
≤10	23(46.00%)	10(58.82%)	
IBIL(μmol/L)			0.971
>14	11(22.00%)	3 (17.65%)	
≤14	39(78.00%)	14(82.35%)	
Albumin(g/L)			**0.009**
≥35	6 (12.00%)	7 (41.18%)	
<35	44(88.00%)	10(58.82%)	
initial treatment status			0.178
prior therapy	20(40.00%)	10(58.82%)	
treatment-naive	30(60.00%)	7 (41.18%)	
TTD^h^, Median (IQR), days	2.50(1.00-6.00)	8.00(2.00-24.50)	**0.012**

Note: a. The single case of Hodgkin lymphoma (n=1) was in the LA-HPC group and was combined with B-cell lymphoma (n=5 in LA-HPC group) for statistical analysis; b. The IPI is a validated prognostic tool for non-Hodgkin lymphoma and is not applicable to the single patient with Hodgkin lymphoma in this cohort. Consequently, the IPI score analysis was restricted to the 66 patients with a diagnosis of non-Hodgkin lymphoma; c. Fever defined as temperature ≥38.5°C; d. Cytopenias defined as affecting ≥2 lineages (hemoglobin <9 g/dL, platelets <100 × 10⁹/L, or neutrophils <1.0 × 10⁹/L);e. Hypertriglyceridemia defined as fasting triglycerides ≥3.0 mmol/L; f. Hypofibrinogenemia defined as fibrinogen ≤1.5 g/L; g. Hyperferritinemia defined as ferritin ≥500 µg/L; h. TTD was calculated as the interval from the date of hospital admission to the date of LA-BMHPC diagnosis. Abbreviations: ALT: alanine aminotransferase; ANC: absolute neutrophil count; AST: aspartate aminotransferase; DBIL: direct bilirubin; ECOG PS: Eastern Cooperative Oncology Group Performance Status; FER: ferritin; FIB: fibrinogen; Hb: hemoglobin; HLH: hemophagocytic lymphohistiocytosis; IBIL: indirect bilirubin; IPI: International Prognostic Index; IQR, interquartile range; LA-BMHPC: lymphoma-associated bone marrow hemophagocytosis; LA-HLH: lymphoma-associated hemophagocytic lymphohistiocytosis; LA-HPC: lymphoma-associated hemophagocytosis without fulfilling HLH criteria; LDH: lactate dehydrogenase; PLT: platelet count; sCD25:soluble CD25 (soluble interleukin-2 receptor); SD: standard deviation; TG: triglycerides; TTD: time to diagnosis.

**Table 2:** Univariate and Multivariate Analysis of Prognostic Factors for Overall Survival

Independent factor	Univariate analysis			Multivariate analysis		
	HR	95%CI	P value	HR	95%CI	P value
Sex	1.001	0.572-1.753	0.996			
Age(years)	1.345	0.750-2.411	0.320			
Pathological type			0.758			
Hodgkin lymphoma and B cell lymphoma^a^	1.000	Reference	NA			
NK/T cell lymphoma	1.153	0.627-2.122				
T cell lymphoma	1.342	0.606-2.970				
ECOG PS	0.964	0.451-2.060	0.925			
Ann Arbor Stage	0.980	0.439-2.188	0.962			
Number of extranodal lesions	1.151	0.589-2.249	0.682			
IPI^b^			0.340			
Low-intermediate risk	1.000	Reference	NA			
High-intermediate risk	2.062	0.600-7.091				
High risk	1.567	0.479-5.128				
Bone marrow involvement	0.829	0.473-1.454	0.829			
Number of involved nodal regions	0.843	0.484-1.467	0.545			
Fever^c^						
Splenomegaly						
Cytopenias^d^	1.765	0.934-3.333	0.080			
Hypertriglyceridemia^e^	1.364	0.696-2.670	0.366			
Hypofibrinogenemia^f^	1.231	0.680-2.230	0.492			
Hyperferritinemia^g^	2.052	0.922-4.564	0.078			
ALT(U/L)	1.216	0.698-2.117	0.490			
AST(U/L)	1.490	0.843-2.635	0.170			
LDH(U/L)	5.877	1.415-24.410	**0.015**	5.991	1.401-25.614	**0.016**
DBIL (μ mol/L)	0.813	0.465-1.422	0.468			
IBIL (μ mol/L)	1.009	0.516-1.971	0.980			
Albumin(g/L)	1.373	0.667-2.829	0.390			
TTD^h^						
initial treatment status	1.803	1.034-3.143	**0.038**	2.537	1.398-4.604	**0.002**
Treatment			**0.003**			**0.001**
HT	0.588	0.226-1.528		0.450	0.172-1.180	
LT	0.268	0.094-0.760		0.138	0.046-0.414	
HLT	0.224	0.090-0.557		0.117	0.069-0.453	
NST	1.000	Reference	NA	1.000	Reference	NA

Note: a. The single case of Hodgkin lymphoma (n=1) was in the LA-HPC group and was combined with B-cell lymphoma (n=5 in LA-HPC group) for statistical analysis; b. The IPI is a validated prognostic tool for non-Hodgkin lymphoma and is not applicable to the single patient with Hodgkin lymphoma in this cohort. Consequently, the IPI score analysis was restricted to the 66 patients with a diagnosis of non-Hodgkin lymphoma; c. Fever defined as temperature ≥38.5°C; d. Cytopenias defined as affecting ≥2 lineages (hemoglobin <9 g/dL, platelets <100 × 10⁹/L, or neutrophils <1.0 × 10⁹/L); e. Hypertriglyceridemia defined as fasting triglycerides ≥3.0 mmol/L; f. Hypofibrinogenemia defined as fibrinogen ≤1.5 g/L; g. Hyperferritinemia defined as ferritin ≥500 µg/L; h. TTD was calculated as the interval from the date of hospital admission to the date of LA-BMHPC diagnosis. Abbreviations: ALT: alanine aminotransferase; AST: aspartate aminotransferase; CI: confidence interval; DBIL: direct bilirubin; ECOG PS: Eastern Cooperative Oncology Group Performance Status; HLH: hemophagocytic lymphohistiocytosis; HLT: simultaneous HLH-directed and lymphoma-directed therapy; HR: hazard ratio; HT:HLH-directed therapy alone; IBIL: indirect bilirubin; IPI: International Prognostic Index; LA-BMHPC: lymphoma-associated bone marrow hemophagocytosis; LA-HPC: lymphoma-associated hemophagocytosis without fulfilling HLH criteria; LDH: lactate dehydrogenase; LT: lymphoma-directed therapy alone; NST: no specific treatment; TTD: time to diagnosis.

**Table 3:** Multivariate Analysis of Therapeutic Components for Overall Survival

Prognostic Model	Variable Analyzed	HR	95% CI	P value
Model A^a^: Impact of HLH-directed therapy	HLH-directed therapy (HT or HLT) vs. No HLH-directed therapy (LT or NST)	0.951	0.517-1.748	0.872
Model B^a^: Impact of lymphoma-directed therapy	Lymphoma-directed therapy (LT or HLT) vs. No lymphoma-directed therapy (HT or NST)	0.301	0.160-0.568	**<0.001**
Model C^a^: Impact of combined therapy	Combined therapy (HLT) vs. Other strategies (HT, LT or NST)	0.630	0.357-1.111	0.110

Note: a. Each model represents a separate multivariate Cox proportional hazards regression analysis, adjusted for LDH level (>245 U/L) and initial treatment status (treatment-naïve vs. prior therapy). Abbreviations: CI: confidence interval; HLH: hemophagocytic lymphohistiocytosis; HLT: simultaneous HLH-directed and lymphoma-directed therapy; HR: hazard ratio; HT: HLH-directed therapy alone; LT: lymphoma-directed therapy alone; NST: no specific treatment.

## Abbreviations

ALT: alanine aminotransferase
ANC: absolute neutrophil count
AST: aspartate aminotransferase
AUC: area under the curve
BMHPC: bone marrow hemophagocytosis
CI: confidence interval
DBIL: direct bilirubin
DEP: liposomal doxorubicin, etoposide, and methylprednisolone
ECOG PS: Eastern Cooperative Oncology Group Performance Status
FER: ferritin
FIB: fibrinogen
Hb: hemoglobin
HLH: hemophagocytic lymphohistiocytosis
HLT: simultaneous HLH and lymphoma therapy
HPC: hemophagocytosis
HR: hazard ratio
HT: HLH-directed therapy alone
IBIL: indirect bilirubin
IPI: International Prognostic Index
IQR: interquartile range
LA-BMHPC: lymphoma-associated bone marrow hemophagocytosis
LA-HLH: lymphoma-associated hemophagocytic lymphohistiocytosis
HPC: hemophagocytosis
LA-HPC: lymphoma-associated hemophagocytosis without fulfilling the HLH criteria
LDH: lactate dehydrogenase
LT: lymphoma-directed therapy alone
MAS: macrophage activation syndrome
M-HLH: malignancy-associated HLH
NST: no specific treatment
OHI: optimized HLH inflammatory index
OS: overall survival
PLT: platelet count
ROC: receiver operating characteristic
sCD25: soluble CD25 (soluble interleukin-2 receptor)
SD: standard deviation
TG: triglycerides
TTD: time to diagnosis
